# Predicting and elucidating the post-printing behavior of 3D printed cancer cells in hydrogel structures by integrating in-vitro and in-silico experiments

**DOI:** 10.1038/s41598-023-28286-9

**Published:** 2023-01-21

**Authors:** Dorsa Mohammadrezaei, Nafiseh Moghimi, Shadi Vandvajdi, Gibin Powathil, Sara Hamis, Mohammad Kohandel

**Affiliations:** 1 Department of Applied Mathematics, University of Waterloo, 200 University Ave West, Waterloo, ON N2L 3G1 Canada; 2 Department of Mathematics, Faculty of Science and Engineering, Swansea University, Swansea, UK; 3 School of Mathematics and Statistics, University of St Andrews, St Andrews, UK

**Keywords:** Cancer models, Biotechnology, Computational biology and bioinformatics

## Abstract

A key feature distinguishing 3D bioprinting from other 3D cell culture techniques is its precise control over created structures. This property allows for the high-resolution fabrication of biomimetic structures with controlled structural and mechanical properties such as porosity, permeability, and stiffness. However, analyzing post-printing cellular dynamics and optimizing their functions within the 3D fabricated environment is only possible through trial and error and replicating several experiments. This issue motivated the development of a cellular automata model for the first time to simulate post-printing cell behaviour within the 3D bioprinted construct. To improve our model, we bioprinted a 3D construct using MDA-MB-231 cell-laden hydrogel and evaluated cellular functions, including viability and proliferation in 11 days. The results showed that our model successfully simulated the 3D bioprinted structure and captured in-vitro observations. We demonstrated that in-silico model could predict and elucidate post-printing biological functions for different initial cell numbers in bioink and different bioink formulations with gelatine and alginate, without replicating several costly and time-consuming in-vitro measurements. We believe such a computational framework will substantially impact 3D bioprinting's future application. We hope this study inspires researchers to further realize how an in-silico model might be utilized to advance in-vitro 3D bioprinting research.

## Introduction

One of the burgeoning 3D biofabrication methods is three-dimensional (3D) bioprinting, which is widely applied in regenerative medicine and tissue engineering to fabricate complex tissue-mimetic structures^1^. The application of this technique has great potential in personalized therapy with more concentration on controlling drug release, drug screening for cancer treatment, studying possible side effects, and analyzing the metastasis and invasion of tumour cells^2^. 3D bioprinting technique combines cells, biomaterials and controlled motor systems to develop complex 3D structures and has accurate control over the structure's features such as mechanical properties, porosity, permeability and stiffness^3–5^. This technique can overcome multiple limitations of the traditional 3D methods by incorporating important aspects of cellular habitat. These aspects include an nonuniform 3D microenvironment similar to the natural extracellular matrix (ECM) of tumours, complex interactions of cells with their neighbouring cells and with local ECM, and complicated diffusion processes of nutrients and oxygen^6–8^. Thus, this method can be used to better represent insights into cell growth mechanisms and provide a closer prediction of in vivo tumour dynamics and cancer cells' response to therapies^7^.

Despite the fast advancement of the 3D bioprinting method, there are some challenges that need to be addressed. Currently, the bioprinting technique is mainly on a trial-and-error basis to achieve the desired output, increasing the need for experimental techniques. This trial-and-error basis includes optimizing bioink properties and its printability, structure mechanical strength, and cell viability during and post-printing. Therefore, it is very costly to optimize bioprinting-related experiments^9^. These challenges make the experimental design and data gathering more complicated in this process.

In silico methods can be used to complement in vitro experiments and assist in addressing some of the limitations of this 3D method^10–12^. Common in-silco approaches include machine learning (ML) methods and mechanistic modelling. ML models can be further categorized as deep neural networks, random forests, support vector machines (SVM), and tree classifiers. ML is becoming more well-known to be applied in different stages of 3D printing process, such as process optimization, construction accuracy analysis, defect diagnosis, and bioink property prediction^13^. For example, Xu et al.^14^ successfully created a predictive model using ML approaches to anticipate cell viability with good sensitivity and to evaluate the importance of various process parameters, including UV intensity and UV exposure time, on cell viability in Stereolithography-based 3D bioprinting. Multiple studies have also tried to develop different ML-based techniques to optimize the printability of bioinks. For instance, Lee j et al.^15^ demonstrated the relationship between printability and the mechanical features of the ink using multiple regression analysis. However, despite the great progress in applying ML models in the biomedical sciences, this method faces some limitations depending on the process of data collection and the study's objective.

To make accurate predictions, ML models require huge volumes of data, selecting a suitable algorithm, and determining inputs and outputs of interest^16^. It may be impractical to acquire large amounts of data from bioprinting research involving costly cells and materials and time-consuming processes. Another common challenge for ML applications is that they may be difficult to interpret. This may lead to a lack of trust from researchers looking for a mechanistic interpretation of cell behavior in bioprinted structures^17^. In contrast, mechanistic modelling concerns describing phenomena via hypotheses for underlying mechanisms. Mechanistic models include stochastic and agent-based models, discrete models, ordinary differential equations (ODEs), and partial differential equations (PDEs). Such mechanistic models may provide insight into the mechanistic aspects of cell behaviors in bioprinted structures. and are, therefore, our preferred mathematical technique in this study.

There is increasing research on mechanistic modelling and simulation as part of the 3D bioprinting process. For example, there are multiple mathematical studies predicting the shear stress applied on the bioink and cells in the nozzle^18,19^; predicting the mechanical properties of the final printed structure based on bioink material properties^20^; and simulating the bioink deposition and the ultimate shape of the created 3D structure^9^. These mathematical techniques have tried to simulate the bioprinting process—during and after bioprinting—to investigate the effect of different parameters on various aspects of bioprinting, including cell viability and structural stability.

**Figure 1:**
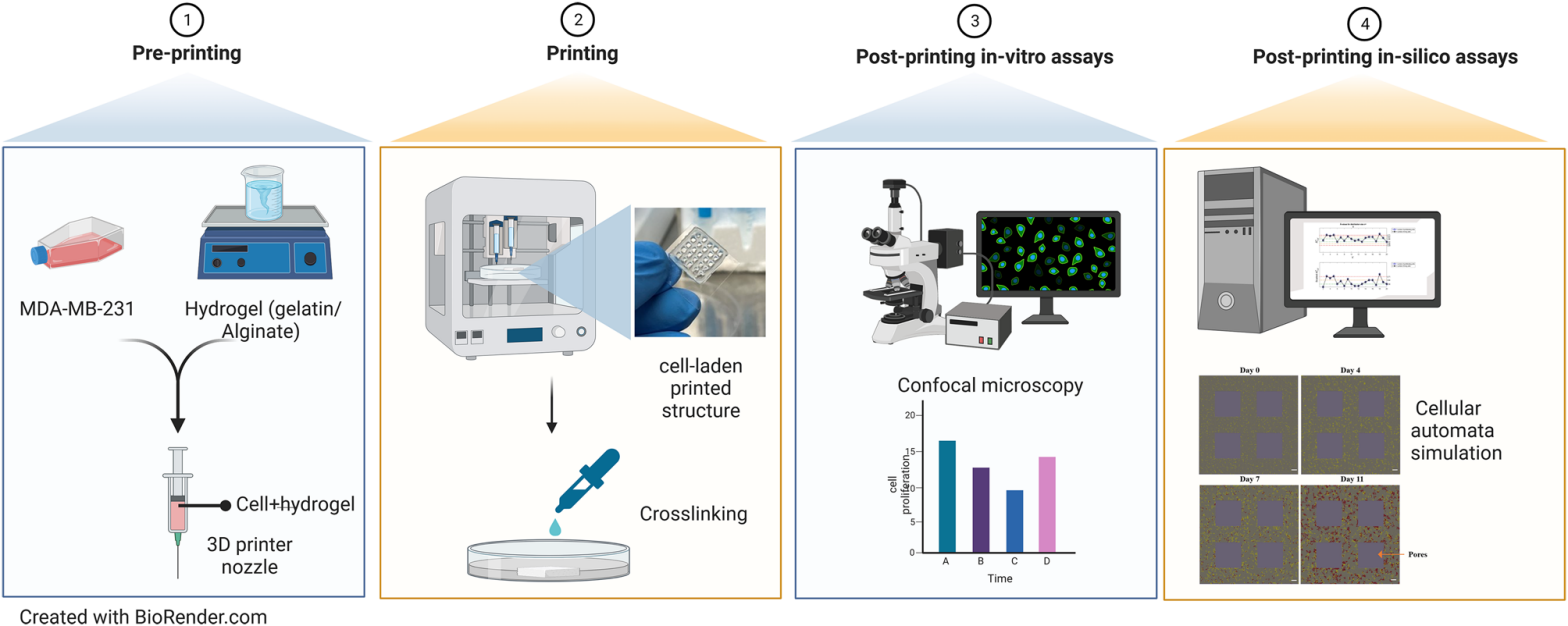
Schematic illustration of the main steps involved in this study; step 1: preparing bioink consisting of gelatine, alginate, and MDA-MB-231 cell line; step 2: printing and crosslinking the cell-laden 3D structure; step 3: performing post-printing in-vitro assays; step 4: developing and calibrating an in-silico model. Created with BioRender.com.

However, A comprehensive computational study of the post-printing behaviour of cells embedded in 3D bioprinted structures is yet to be reported. Such mathematical studies can provide researchers with more insights into post-printing cell functioning and allow them to predict complex cellular activities and optimize the cellular microenvironment to save money and time by keeping experimental evaluations to a minimum. This is the first study that aims to develop an integration of in-vitro and in-silico 3D bioprinted breast cancer models to study the post-printing behaviour of a population of breast cancer cells embedded in a 3D bioprinted structure (Fig. 1). In the in-vitro study, we used the MDA-MB-231 cell line, one of the most aggressive breast cancer cell lines most frequently used in cancer-related studies. To develop the bioink, a mixture of gelatin and alginate is selected as the most common bioink material in 3D bioprinting due to its similar characteristic to native ECM and appropriate physiological and biological properties for bioprinting application^21^. For our in-silico study, we selected cellular automata modeling, in which most of the parameters were picked from experimental data. A comprehensive in-vitro assays were also done to improve the reliability of the model.

The proposed agent-based model in this study is beneficial for demonstrating complex cellular systems, including cellular proliferation, movement, cell interactions with the environment (e.g., ECM, neighbouring cells and resource consumption) and cell aggregation within the scaffold. In this study, we demonstrate that mathematical models and in-silico simulations can be used to capture in-vitro dynamics. Such a combination of in-vitro and in-silico studies can improve researchers' understanding of cell activities within 3D bioprinted constructs with the purpose of accelerating and increasing the accuracy of optimization and experimental settings. The proposed in-silico model is very promising and is able to be further developed to be applied in 3D cell culturing using bioprinting techniques in different biomedical applications.

## Result and discussion

### In-vitro cell studies

**Figure 2:**
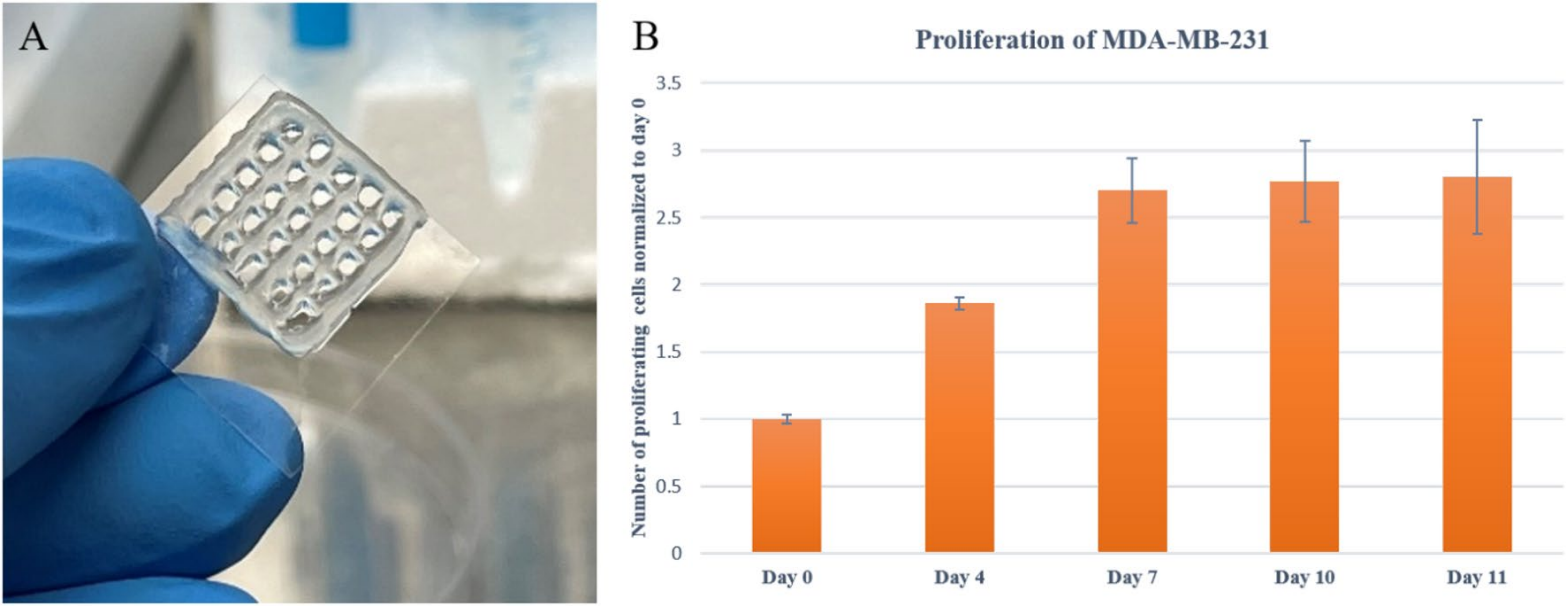
(**A**): Fabricated cell/hydrogel structure using 3D bioprinting technique; (**B**): MDA-MB-231 proliferation embedded in hydrogel network post printing from day 0 to day 11. Error bars represent ± SD, n ≥ 3.

The tumour-like hydrogel network model was successfully printed (Fig. 2a). To determine the proliferation of MDA-MB-231 cells embedded within the hydrogel, MTT assay was used to monitor cellular metabolic activity on days 0, 4, 7, 10, and 11. As illustrated in Fig. 2b, compared to day 0, MDA-MB-231 cells in the 3D cell/hydrogel construct demonstrated 1.86-, 2.7- and 2.78- and 2.8- fold proliferation on days 4, 7, 10, and 11, respectively. Indeed, cells showed rapid proliferation in the first 7 days and almost maintained almost a plateaued cell proliferation from day 7 to 11. Interestingly, the results showed that the doubling time of the MDA-MB-231 encapsulated in the 3D microenvironment was three times higher than that of cells grown in 2D cultures, which can be attributed to the reduced cell activities within the 3D matrix^22^. From day 7 to day 11, the number of proliferating cells remained approximately constant and left a question behind: whether cells died or entered to non-proliferating phase (quiescent) due to undesired conditions. To answer this question, live-dead assay, as well as Ki-67 immunostaining, were further employed during 11-days period to get a better understanding of cell behaviour growth encapsulated in the 3D scaffold.

The viability of MDA-MB-231 over 11 days was visualized using live-dead staining, which was in line with MTT assay results. As shown in Fig. 2, most cells were viable after printing, and the viability rate of cells was 76 ± 2% on day 0, which clearly demonstrated minor damage of the bioprinting process on cell viability. The rate of viability increased over the first week and reached 98 ± 1% and 99 ± 1% on day 4 and day 7, respectively. Therefore, the structure was porous enough for oxygen and glucose to diffuse and distribute through the hydrogel scaffold, which could provide a proper living environment for cells. From day 7 to day 11, although some cells died, the majority of cells survived, and the rate of viability reached 96 ± 2% on day 11. By comparing the results of live-dead assay and MTT assay, it can be concluded that a significant portion of cells entered the resting phase after seven days since the maximum capacity of the scaffold had been achieved, and some of the cells began to die during the long-term stationary phase, or due to the lack of resources. Cell death in this experiment is almost negligible, which illustrates the promising potential of gelatin/alginate scaffold for bioprinting applications for a long time.

**Figure 3:**
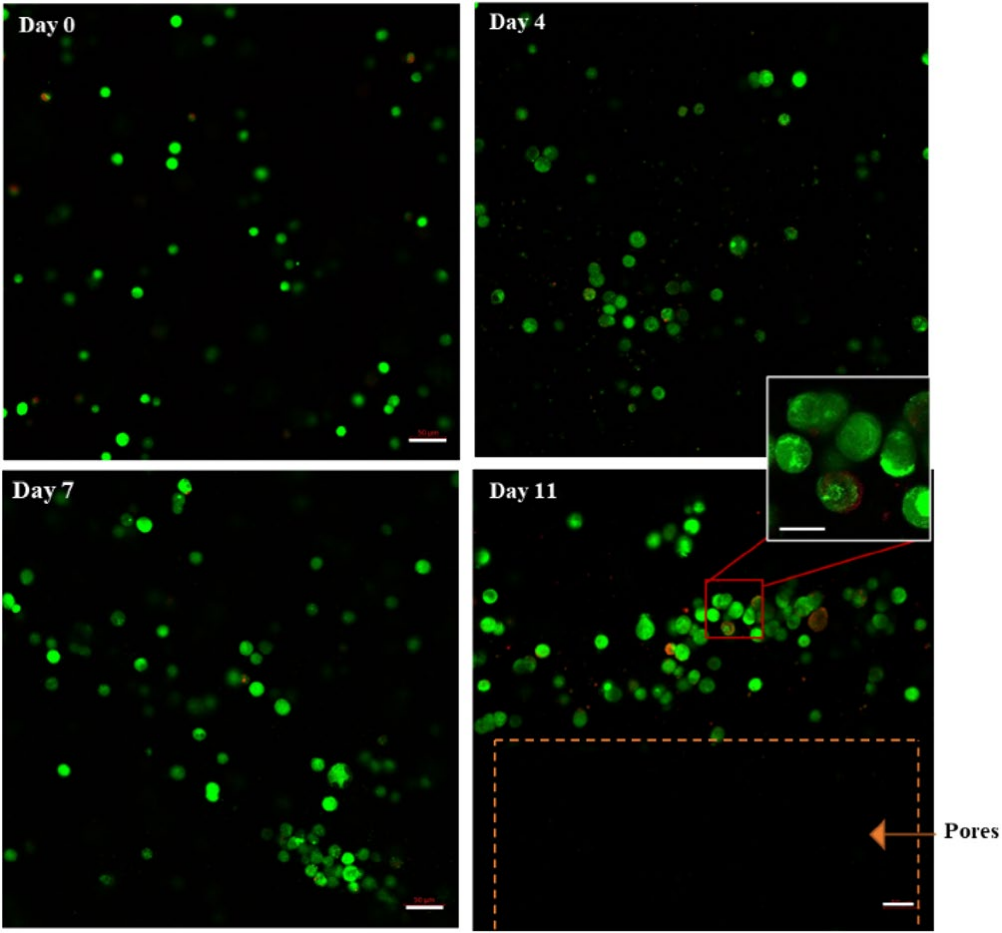
Microscopic images demonstrating viability of MDA-MB-231 cells within 3D hydrogel constructs using the fluorescent live/dead assay kit from day 0 to day 11. Live and dead cells were stained using calcein-AM and PI, respectively (green color represents live cells; red color represent dead cells). Cells were imaged using the laser scanning confocal microscope. Scale bar, 50 μm (enlarged images, scale bar, 30 μm).

**Figure 4:**
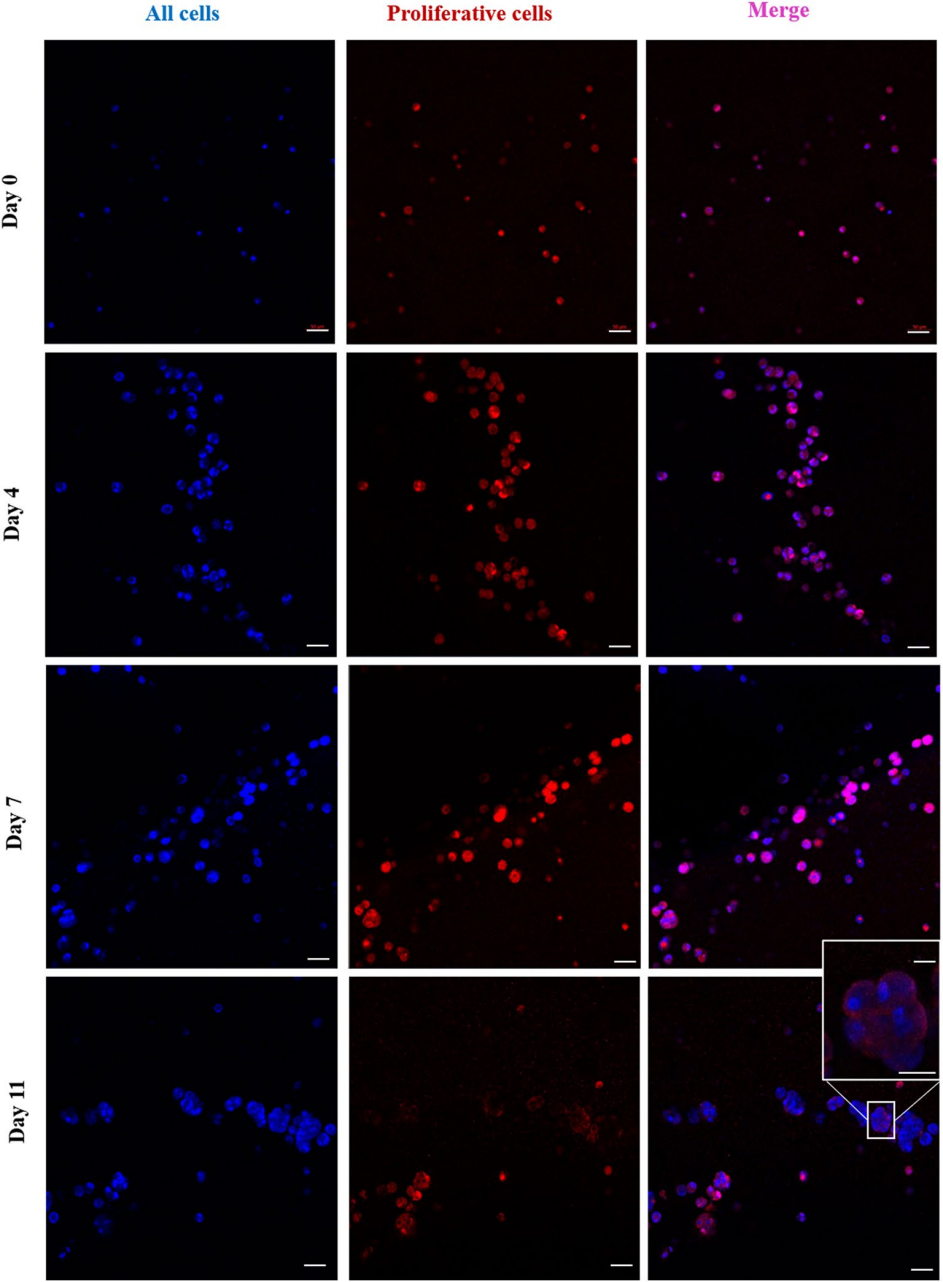
Ki-67 staining of encapsulated MDA-MB-231 cells within 3D bioprinted constructs. Cells were stained using anti-Ki-67 antibodies visualized with Alexa Fluor 546, and Hoechst 33,342 (red color represents cells positive to ki-67; blue color represents all cells). Cells were imaged using the laser scanning confocal microscope. Scale bar, 50 μm (enlarged image, scale bar, 30 μm).

To visualize the proliferative capacity of MDA-MB-231, cells were fixed, and an anti-Ki-67 antibody was used to image proliferating cells using a confocal microscope (Figs. 3, 4). Ki-67 is a common-used marker that is present for all active phases of the cell cycle but absent in cells at the stationary phase^23^. The results demonstrated that on days 0 and 4, almost 98 ± 1% and 95 ± 2% of the cells were positive for ki-67, respectively, while this number decreased to 86 ± 2% on day 7, followed by a dramatic drop to about 48.2 ± 2.4 on day 11. This result is in agreement with the data in the previous (Fig. 3), illustrating that within seven days, cells not only survive but also maintain their proliferating ability. From day 7 to day 11, although a high proportion of cells demonstrated to be alive, they were quiescent and were not able to proliferate anymore because of the lack of enough space for cells to proliferate. Additionally, on days 7 and 11, cells became more aggregated, particularly close to the pores, and cells at the center of the cell aggregates were shown to be non-proliferating due to being surrounded by other cells. Therefore, the proliferation process was aborted as there was not enough space for daughter cells to be placed and due to the lack of nutrients and oxygen at the center of aggregates.

The results of the in-vitro experiments were applied to parameterize the developed mathematical model.

### In-silico cell studies

An essential property that makes the 3D bioprinting technique superior to the other 3D cell culture techniques is the precise control it offers over created structures. This property provides the opportunity to fabricate bio-mimetic structures with controlled structural and mechanical properties such as porosity, permeability and stiffness with high resolution^24–26^. However, analyzing post-printing cell behaviour within the scaffold depends only on performing different in-vitro measurements. Experimentally measuring some of aspects of cellular behaviour in 3D bioprinted structures is challenging due to due to the lack of precise quantitative techniques, such as defining cell–cell and cell-microenvironment interactions. This problem has motivated our development of a mathematical framework to simulate post-printing cell behaviour within the scaffold. This framework must not only overcome these challenges but also accurately designs and predicts post-printing cellular functions without replicating experiments.

Individual-based modelling using a cellular automaton is one method to simulate the spatial and temporal mechanism of cell growth at the cellular level^27,28^. This approach is a dynamic system that includes grids of cells, and each cell has sets of discrete states^29^. CA modelling has been widely used in recent years to investigate different cancer cell mechanisms based on a variety of static automaton rules^30^. However, to date, no study has applied CA modelling in 3D culturing cancer cells using 3D bioprinting. We selected this mathematical method for this study on simulating cell growth encapsulated in a 3D bioprinted structure due to its ability to capture spatial properties of the 3D printing structure and its flexibility to explore different hypotheses. Additionally, since the data obtained from our in-vitro experiments contained cells in discrete form, this discrete mathematical technique would have a more accurate simulation of this process. The framework developed in this study represents rules in cellular proliferation, viability, movement, and interactions with the environment, which includes hydrogel and neighbouring cells, as well as cluster formations within the hydrogel network. The in-silico results demonstrated in this article are based on mean values, and standard deviations from n = 100 simulation runs, where n is motivated by consistency analysis (Supplementary Material, Consistency Analysis).

**Figure 5:**
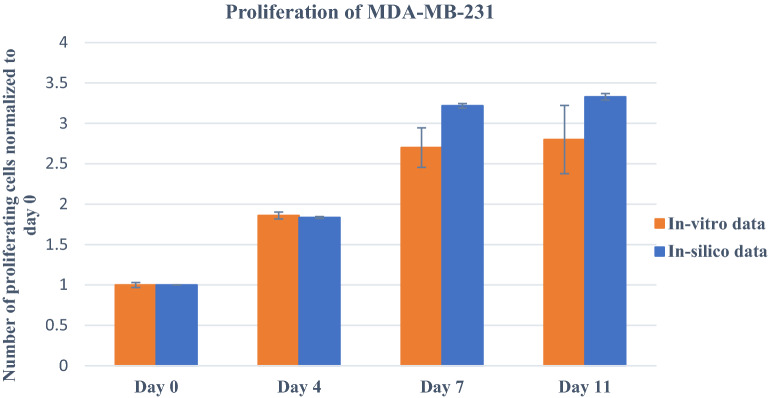
Comparison between in-vitro and in-silico results of MDA-MB-231 cell proliferation within the 3D hydrogel network. Error bars represent ± SD, n ≥ 3.

Figure 5, showing the cell proliferation patterns for 11 days within the scaffold for both in-vitro and in-silico, illustrates them in agreement with each other. In cell proliferation, the initial cell density and scaffold capacity are two key parameters that we specified as ${C}_{\mathrm{initial}}$ and $C$ variables, respectively. The corresponding values of these parameters were determined using calibration to produce the best fit to the in-vitro studies. Note that, the initial cell density in the developed model represents the effective initial population of cells that can interact with one another within a thin layer, not the total number of cells in the scaffold. Therefore, the simulated cell density was reduced by a scale factor compared to the cell density in the experimental settings. The results showed that cells reached the maximum cell density of cells in both simulation and experiments after seven days. The time it took for cells to achieve maximum density in the scaffold was strongly dependent on the number of initial cells and the capacity of the printed scaffold. The greater the number of initial cells and the less the scaffold capacity, the sooner cells reached maximum cell density and stopped proliferation. Therefore, by fine-tuning these parameters and running simulations in different scenarios, researchers can design the experiments to achieve desired results without repeating in-vitro assays.

Simulated data were also able to consistently replicate the viability and proliferation experimental results. As explained in the previous section, although cells showed around 99% viability within seven days of printing, the number of dead cells slightly increased from day 7 to day 11 when the significant portion of cells was in the resting phase. Hence, to precisely simulate the in-vitro condition, it was assumed that cells that remained in a prolonged stationary phase for more than the specified hours, defined by a stochastic number (${C}_{d}$), started to die with a specified probability (${P}_{d}$). This observation can be explained biologically by the cells' inability to re-enter the cell cycle after entering the cell stationary phase.

Figure 5 shows the snapshots of in-silico MDA-MB-231 cells growing within the hydrogel network; as well as in-vitro microscopic images of cells in the 3D structure. In this figure, you can see cells' distribution and progression of cell cluster formation on days 0, 4, 7 and 11 for both in vitro and in silico. In the in-silico images, yellow, red and black coloured cells are representative of proliferating, non-proliferating and dead cells, respectively.

On day 0, a few cells were distributed inside the hydrogel network. Over time, cells proliferated and created the first two-cell clusters and then bigger ones. Similar to in-vitro observations, the percentage of viability remained around 100% until day 11, when the viability slightly decreased and reached 93.74 ± 0.5%. Furthermore, simulated cell proliferation decreased over time and after seven days experienced a significant drop due to achieving the maximum capacity of the scaffold; and finally dwindled to 54.14 ± 0.25% on day 11. The animation of cell growth within the hydrogel scaffold in 11 days is also available in the Supplementary Material (Figure S3).

Another important factor apart from cell viability and proliferation is the ability of the cells to move in their surrounding matrix. This simulation can also be applied to analyze the cell movement as well as the structure and distribution of formed tumour clusters in the hydrogel network without experimental assessment. Tumour clusters might be created due to interactions between neighboring cells or between parent and daughter cells, depending on their position and the microenvironment^31^. Indeed, cells coordinate through cell–cell physical and signalling interactions and create clusters.

**Figure 6:**
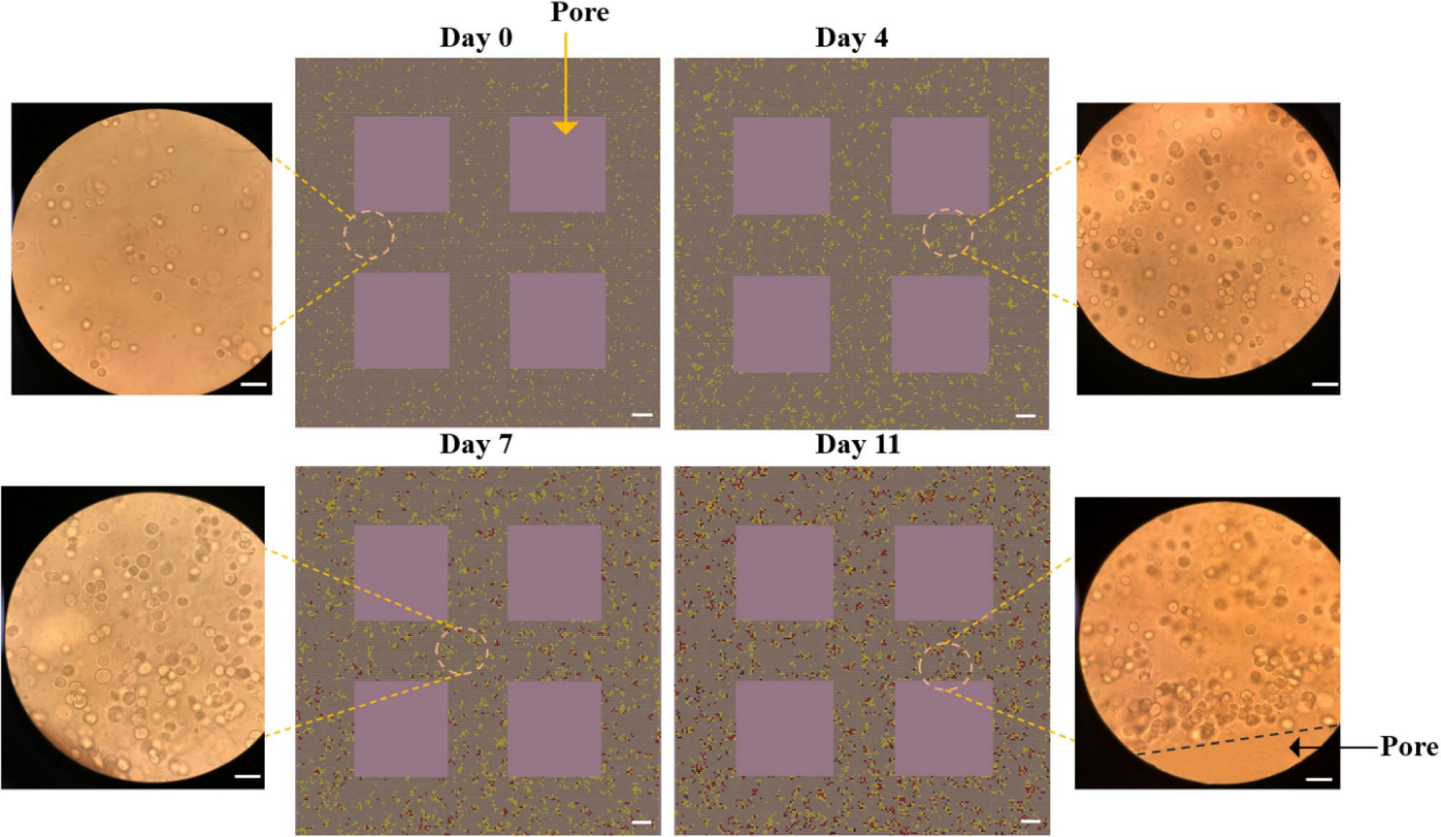
Middle panels visualize the MDA-MB-321 growth within the 3D hydrogel construct in silico; yellow represents proliferating cells; red represents non-proliferating cells; black represents dead cells. The right and left panels represent MDA-MB-231 cells encapsulated in 3D hydrogel constructs observed by a phase-contrast microscope on day 0, day 4, day 7, and day 11: scale bar, 50 μm.

In Figure S1 (Supplementary Material), cells show a trend of crawling toward scaffold pores followed by forming clusters around those pores, as essential resources are in greater concentration there, particularly after seven days. This fact suggests that the hydrogel networks had limited resources transport capacity. Thus, we have defined particular ranges of attraction for both cell–cell signalling (${L}_{C}$) and cell-pore attraction (${L}_{p}$) to consider different cell migration directions. Speed of movement was another important parameter affecting cluster formation. Fallica et al. illustrated that the movement of cancer cells is inhibited in 3D microenvironments and shows extremely low speed due to the combination of material rigidity and the anchoring of cell receptors^22^. Therefore, based on the observations in this and previous studies, we have calibrated cell movement speed. In the movement processes, each individual moved at a specific time defined as ${m}_{C}$ in a direction determined based on cell attraction. During the process of calibration, by comparing in-vitro and in-silico results, it was concluded that cells had less tendency to move toward neighbouring cells within a smaller attraction range (${L}_{C}$) compared to the surface/pores of the scaffold (${L}_{p}$). Applying these rules in the model (Fig. 6), cells mimic the in-vitro cellular behaviour in terms of movement and cluster formation. The results of the proposed model are consistent with those of previous studies^22,32^.

In general, this model is developed to combine with in-vitro 3D-bioprinting evaluations, leading to a comprehensive analysis of the whole 3D fabricated structures. One of the main applications of this simulation is to predict the post-printing cellular behaviour in an unpracticed microenvironment which improves its capability to replicate desired biological settings. For example, this model provides the opportunity to evaluate the impact of different important parameters such as various initial cell densities on cellular behaviour in a long-term period. This can be of benefit to researchers to generate a more suitable microenvironment for cell growth without the need to repeat experiments. For instance, they can design the scaffold in terms of size or structural shape with the purpose of modifying the scaffold capacity to improve cell proliferation and decrease cell death.

### In-silico model validation

To further validate the in-silico model, we performed the bioprinting procedures with different experimental variables in two different situations: case 1: varying initial cell densities; case 2: varying bioink formulation. In case 1, we did bioprinting with a bioink with 4% ($w/v$) gelatin, 4% ($w/v$) alginate, and 1.5 × 1 ${0}^{6}$ MDA-MB-231 cells ${\mathrm{mL}}^{-1}$. In case 2: we performed bioprinting experiments with a bioink with final concentrations of 4% ($w/v$) gelatin, 5% ($w/v$) alginate, and 2 × 1 ${0}^{6}$ MDA-MB-231 cells ${\mathrm{mL}}^{-1}$. Using the calibrated in-silico model, we would like to predict the proliferation pattern of cells in these two new conditions.

**Figure 7:**
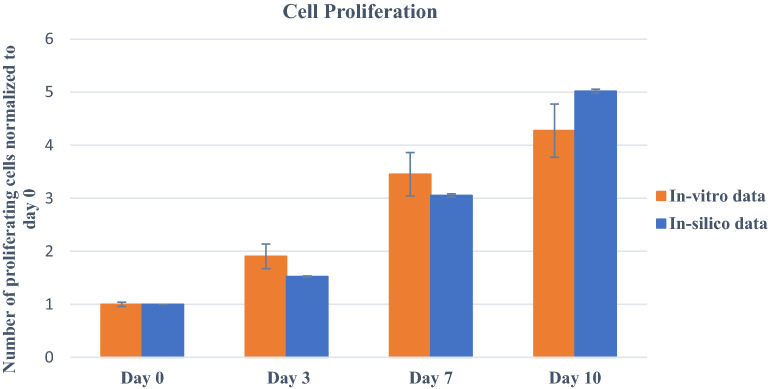
Prediction of cell proliferation using in-silico model for case 1: 1.5 × 1 ${0}^{6}$ MDA-MB-231 cells ${mL}^{-1}$ in 4% gelatin/4% alginate bioink; on day 0, 3, 7, and 10. In vitro study was performed using the MTT assay. Error bars represent ± SD, n ≥ 3.

Figure 7, showing the cell proliferation patterns for 10 days for case 1 for both in-vitro and in-silico, illustrates them in agreement with each other. In the in-silico model, all parameters except ${C}_{\mathrm{initial}}$ (the initial cell density) have the same values as in the calibrated model. The simulated cell density is reduced by the same scale factor compared to the cell density in the experimental settings and set to ${C}_{\mathrm{initial}}=2000$. Both simulations and experiments demonstrated that cells did not achieve the maximal cell density after 7 days and kept growing. Hence, when the number of beginning cells reduced, the later cells attained their scaffold capacity and consequently ceased proliferating.

**Figure 8:**
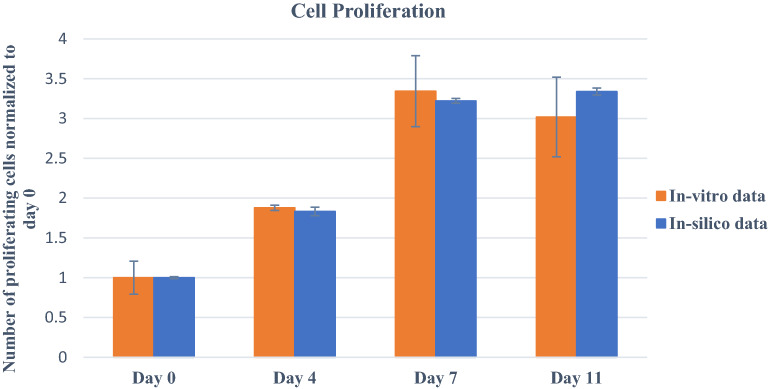
Prediction of cell proliferation using in-silico model for case 2: 2 × 1 ${0}^{6}$ cells ${mL}^{-1}$ in 4% gelatin/5% alginate bioink. In vitro study was performed using the MTT assay. Error bars represent ± SD, n ≥ 3.

Figure 8, comparing in-vitro and in-silico cell proliferation patterns for case 2, also shows agreement. In this case, experimentally, we altered the formulation of bioink. Increasing the alginate concentration can increase the rigidity of a hydrogel-based construction, as demonstrated earlier^33^. It has also been found that the stiffness of the microenvironment would also affect cell movement and spheroid formation within the scaffold^34^. Although parameters directly related to stiffness have not yet been integrated into our model, we may regulate cellular behavior and investigate the impacts of bioink formulation and stiffness on proliferation and migration by varying some rules of cellular movement. In the calibrated model for bioink containing 4% (w/v) gelatin and 4% (w/v) alginate, cells move every 15 h, denoted by ${m}_{c}$. Thus, with 4% (w/v) gelatin, 5% (w/v) alginate-based bioink, we reduce the movement speed of cells encapsulated in a stiffer microenvironment and change ${m}_{c}$ to 20 h while keeping other parameters unchanged. Comparing the results of an in-silico model to in-vitro data, we conclude that for 4% (w/v) gelatin and 5% (w/v) alginate-based bioink, ${m}_{c}$=20 h closely match the in vitro proliferation trend within 11 days.

The in vitro observations revealed that on day 11, cell proliferation decreased slightly, which can be explained by the stiffer microenvironment. Indeed, the rigidity might reduce cell movement and proliferation; and hinder the transport of nutrients, resulting in cell death over time. For more significant changes in bioink formulation, it is necessary to incorporate microenvironment stiffness or bioink-related parameters into the model to anticipate cell behaviour accurately. However, with 4% ($w/v$) gelatin, 5% ($w/v$) alginate, and minor modifications to the bioink formulation, our developed model can be used successfully.

Taken together, we could validate our model by creating variations in the in vitro data and successfully simulating the varied situation. Hence, we can confidently claim that this model can help researchers plan experiments more accurately by predicting the outcome. In fact, researchers executing simulations under various situations and fine-tuning related parameters can design experiments to reach the desired results without repeating in-vitro procedures.

### Prospect

The developed mathematical framework in this study can be extensively applied in different bioprinting-related studies for various applications such as tissue engineering, oncology, and the pharmaceutical industry by extending its rules and improving its ability to provide an accurate prediction of biological systems. Our model can be expanded by incorporating bioink-related parameters such as stiffness and structural integrity, which regulate cellular behaviour, including proliferation and migration and oxygen/nutrient diffusion to the 3D network^35–39^.

Moreover, the proposed model can be integrated with the machine learning algorithms and provide researchers with this opportunity to predict the temporal or structural effect of the hydrogel network on any desired objectives in the biological system. Furthermore, we can use CA simulation to pre-train the ML algorithm and then a transfer learning approach can be applied to train the experimental data.

Another prospect of this model is its application in a heterogeneous environment with multiple cell lines for studying cell–cell interaction and cell-ECM interactions. Besides, by adjusting the rules, this model can be integrated with pharmacokinetic modelling techniques to simulate drug treatment responses in 3D cell culture using 3D bioprinting to help study tumour development and metastasis, drug screening, and other aspects of cancer research.

In the end, we believe that this work or its combination with other modelling techniques can significantly influence the development of 3D bioprinting in the future and avoid conducting costly and time-consuming experiments to a great extent.

## Conclusion

So far, optimal post-printing behaviour of cells growing within the 3D biorinted hydrogel has been achieved using only replicating several experiments, which are time-consuming and expensive. In an attempt to overcome this challenge, we developed a CA model to simulate post-printing cells' dynamics within the 3D bioprinted construct. To this end, we first successfully printed MDA-MB-231/gelatin/alginate bioink and evaluated cellular behaviour in 11 days using MTT, Live-dead and Ki-67 cell proliferation assays. Using in-vitro results, we defined rules in the CA model for cell proliferation, viability, movement and cluster formation within the 3D hydrogel network and calibrated model parameters such as doubling-time, movement speed, and probability of death in 11 days. Our model can quantitatively capture the post-printing in-vitro behaviour of cells in the 3D scaffold and is able to predict and elucidate the cell behaviour for different bioprinting conditions. For example, it replicates the cellular movement and cell crawling toward the pores, followed by forming clusters after seven days due to uneven distribution of nutrients and oxygen. Furthermore, the in-silico data elucidate the dependence of cell proliferation on the initial number of cells and the capacity of the printed hydrogel network, and can predict post-printing cellular proliferation based on the initial quantity of cells in bioink. This in-silico model can also represent cell activity using various network formulations containing gelatin and alginate. The proposed mathematical framework can be of benefit to researchers to generate a more suitable microenvironment for cell growth without the need to repeat experiments. Hence, our mathematical framework can be considered a resourceful step involved in the advancement of bioprinting-related studies.

## Materials and methods

### Materials

The alginic acid Sodium salt, dimethyl sulfoxide (DMSO), Sodium chloride, Calcium chloride (CaCl_2_) and gelatin from bovine skin (type B) were obtained from Sigma-Aldrich (Canada). For cell culture studies, MDA-MB-231 were purchased from ATCC, DMEM (Dulbecco's Modified Eagle Medium), FBS (fetal bovine serum), penicillin/streptomycin, Trypsin/EDTA solution at 0.25% (w./v.) and phosphate buffer saline (PBS) tablets were bought from Wisent Bioproducts. (3-(4,5-dimethyl-2-thiazol)-2,5-diphenyl-2H-tetrazolium bromide) MTT powder, Triton X-100, BSA (bovine serum albumin) and paraformaldehyde were bought from Sigma-Aldrich (Canada). Furthermore, a Live/Dead Cell Viability assay kit (CBA415), Hoechst 33342Nuclei Dye were provided from Sigma-Aldrich (Canada). Additionally, Anti-Ki67 antibody (ab15580), Alexa Fluor 546 Goat anti-Rabbit IgG (H + L) were purchased from Abcam and Invitrogen (Canada), respectively.

### Cell culture

MDA-MB-231 cells were cultured in DMEM supplemented with 10% FBS and 1% 100 Uml − 1penicillin/streptomycin in T-75 flasks. Cells were incubated at 5% CO2 and 37 °C, and the media was exchanged every other day. When cells reached 80% confluency, the cells were rinsed twice with DPBS and then suspended with trypsin/EDTA (0.25%-1X).

### Bio-printing

To prepare bioink, according to the protocol provided by Ouyang et al.^38^ first 0.5% NaCl solution using DI water was prepared. Then, gelatin and alginic acid sodium salt powder were added, and the solution was stirred vigorously for 1 h. The prepared solution was sterilized by heating at 70 °C (30 min, three times) and then kept at 4 °C before usage. Prior to bioprinting, MDA-MB-231 suspension was gently and evenly mixed with prepared gelatin/alginate solution using a mixing syringe to make a bioink with final concentrations of 4% ($w/v$) gelatin, 4% ($w/v$) alginate and 2–2.5 × 1 ${0}^{6}$ MDA-MB-231 cells ${\mathrm{mL}}^{-1}$.

Bioprinting was carried out using CELLINK INCREDIBLE + extrusion-based bioprinter. The prepared bio-ink was extruded using a needle to fabricate the 3D cell-hydrogel layered grid constructs. More specifically, the printing nozzle applied for this experiment was a standard conical nozzle (22G), and the moving speed of the needle was adjusted at 5 $mm{s}^{-1}$. The printing was done at room temperature by applying appropriate pressure. The structure was designed using Solidwork software and sliced in sli3r software to 10 layers with the size of $10\times 10\times 3 m{m}^{3}$ with a rectilinear filling pattern. During the printing, we made an effort to maintain the pressure at the least level to have the minimum damage to cell viability. Subsequently, the cell-laden constructs were immersed in 3% ($w/v$) sterile calcium chloride solution (20 min) for cross-linking sodium alginate with calcium ions^21^. Then, after being washed with PBS three times, each structure was cultured in DMEM medium containing 10% FBS and 1% penicillin/streptomycin in a 12-well plate and incubated at 5% CO2 and 37 °C for a predetermined period of time. Culture medium was exchanged every other day.

### MTT assay

Cell proliferation within the 3D network was assayed using MTT assay. After predetermined days of incubation, the medium in each well of 12-well plate containing a 3D structure was removed, and 900 $\mu L$ fresh media and 100 $\mu L$ of MTT solution (0.5 ${\mathrm{mg }mL}^{-1}$ in PBS) was added into each well, and the plate was incubated for 4 h at 37 °C in a ${\mathrm{CO}}_{2}$ incubator. The control was a 3D structure without any cells. Then, after aspirating the medium, 1 $mL$ of DMSO was added into each well to dissolve formed formazan crystals for 30 min at 37 °C in a ${\mathrm{CO}}_{2}$ incubator. In the end, the intensity of the solubilized formazan crystals was recorded using the Absorbance Microplate Reader at 540 nm.

### Live-dead assay

The 3D bioprinted cell-laden constructs were stained at specific time points using Live/Dead staining viability kit based on the manufacturer's instructions to determine cell viability. Briefly, each construct was washed in PBS three times. Then, 1 $\mu M$ Calcein-AM and 2 $\mu M$ propidium iodide were applied to stain cells while incubated in darkness. A laser scanning confocal microscope (Zeiss LSM 700) was used for imaging the live and dead cells within the 3D constructs at multiple spots. Then, images were analyzed using ImageJ software. Cell viability was counted by dividing the number of green (living) cells by the total number of cells in each image.

### Cellular proliferation

Ki-67 Cell Proliferation Assay is a quantitative technique to evaluate cell proliferation in vitro. Using this method, the Ki-67 protein is applied as a marker for cell proliferation as it is expressed during the active cell cycle (G1, S, G2, and M) but not the stationary phase (G0). For doing this essay, first, the medium of each well at predetermined timesteps was removed entirely, and each scaffold was washed two times with PBS. Then, cells were become fixed by incubating scaffolds in 4% paraformaldehyde in PBS for 1 h at room temperature. Fixed structures were permeabilized in PBS containing 0.1–0.25% Triton X-100 for 15 min after being washed with PBS two times. Afterward, cells were blocked with 3% BSA/0.1% Triton X-100 in PBS for 1 h before the addition of Anti-Ki67 antibody with the concentration of 5 ${\mathrm{\mu gmL}}^{-1}$ in PBS/1% BSA and incubation overnight at 4 °C. Then, the 3D structures were washed three times for 5 min with PBS/1% BSA prior to the addition of the Goat anti-Rabbit IgG (H + L) Secondary Antibody and incubation for 2 h. Next, the structures were rewashed (two times, 5 min) with PBS/BSA before adding Hoechst 33,342 (incubation of 1 h). Ultimately, the cells were imaged using the laser scanning confocal microscope.

## Computational methodology

### Cellular automata model description

**Figure 9:**
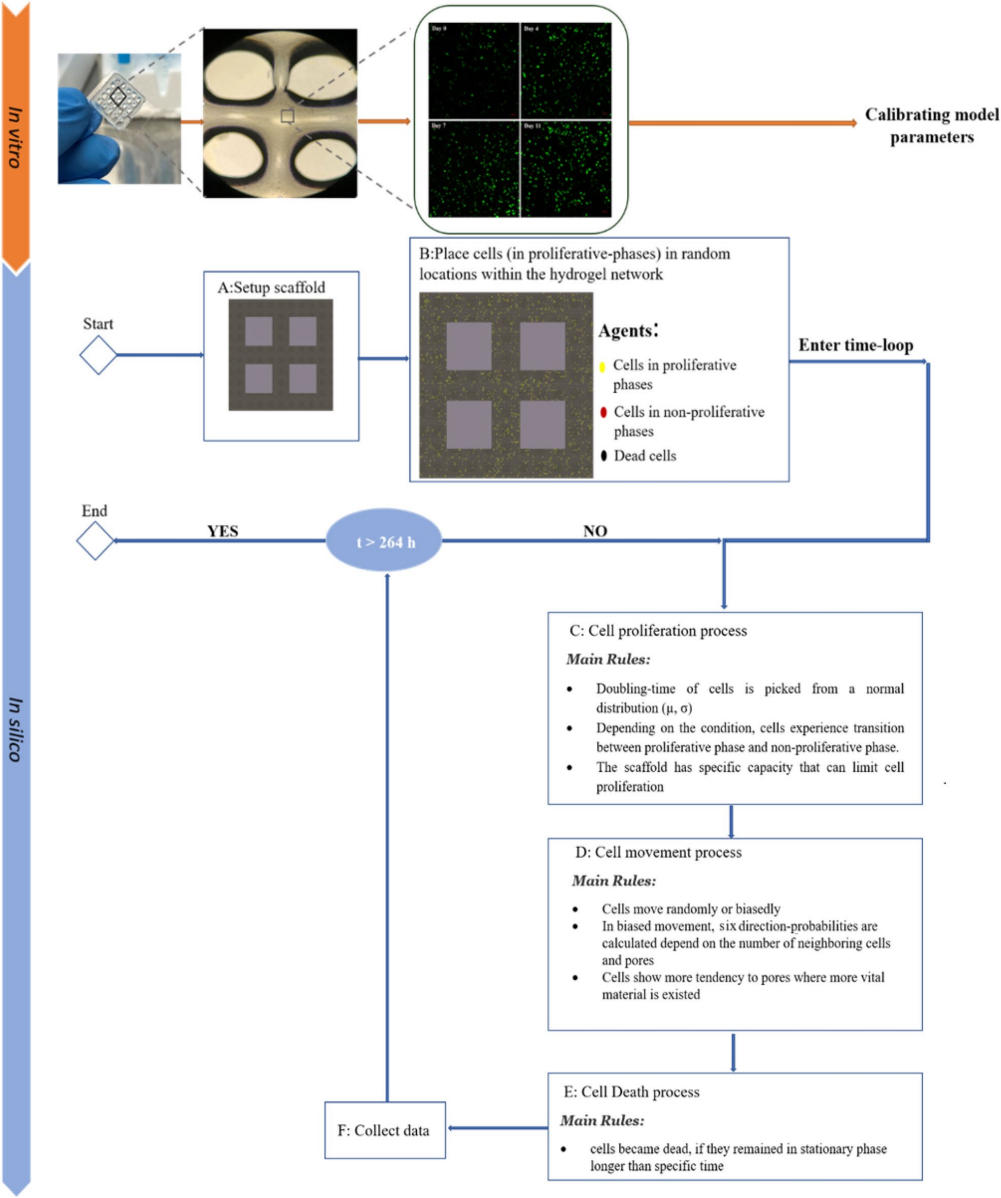
Main steps in in-vitro and in-silico bioprinting.

This model simulates time as discrete, uniform time steps; each time step is 1 h, and each simulation lasts for 11 days. A subdomain of the porous cell-laden scaffold fabricated using the 3D bioprinting method is simulated in this model, which comprises a square lattice of 190 $\times$ 190 $\times 30$ lattice points, symmetrically consisting of four pores with widths of 50 $\times$ 50 $\times 30$ lattice points. Each lattice point within the hydrogel can be occupied by a cell or remain vacant, while the grid points in the pores should remain unoccupied, as the cells in the corresponding in-vitro experiments do not move into the pores (Supplementary Material, Figure S2). A simulation is instantiated by placing a specified initial number of cells at random locations on the lattice within the hydrogel. At each time step, cells behave according to a set of stochastic rules that describe cellular processes such as proliferation, movement, and death. The main algorithm and schedule of processes within each time step in bioprinting are depicted in Fig. 9. The computational framework builds upon previous theoretical cell population work^40,41^. A detailed model description, formulated using the Overview, Design concepts and Details (ODD) protocol, is provided in the Supplementary Material (Supplementary Material, In-silico studies).

### Cell proliferation process

This process is simulated for each cell individually to consider variations among cells. Individual cells are characterized by a specific, stochastic doubling-time, which attributes to the time it takes for each cell to complete one cell cycle to divide. Based on our experimental data, the doubling-time for each cell is picked from a normal distribution with a value µ = 96 h and a standard deviation σ = 6 h. The modelled cell cycle process in proliferation consists of transitions between proliferative and non-proliferative (G0) phases. After dividing, parent cells remain at their current position, and daughter cells can be placed in an unoccupied lattice point in the neighbourhood of the parent. If every neighbouring site is already occupied, parent cells enter the G0-phase, known as the resting or quiescence phase, where cells become inactive^42^. First, second and third-order Moore neighbourhoods are used for placing daughter cells. The scaffold has a specified maximum carrying capacity $C$ to accommodate cells, and upon reaching the total number of cells to this capacity, the proliferation aborted with a specified probability (${P}_{0}$). The carrying capacity depends on spacial and nutrient limitations in the in-vitro system. The values of $C$ and ${P}_{0}$ are calibrated according to the in-vitro observations. Due to the unequal distribution of nutrients and oxygen within the scaffold, some cells can still proliferate after reaching the scaffold's carrying capacity, while others enter the G0 phase. This means that cells can continue to proliferate at sites with sufficient nutrients and free neighbouring lattice points. Table S1 contains the values for all in-silico parameters.

### Movement process

In movement process, cells move every specific time known as ${m}_{c}$, however as all the cells might not be synchronized in their movement, a random number was introduced to be added to ${m}_{c}$. The value of the ${m}_{c}$ parameter was also calibrated using in-vitro data. In this process, individual cells can move in a random manner with random-probability, or biased-random manner with biased-probability, and change their positions if there is a free lattice point in their neighbourhood^34^. Cells can move toward one of six directions (right, left, up, down, forward, and backward) of their neighbourhood with equal probability, which is known as random movement, or move in a biased-random manner with weighted probability, where cells are attracted to other cells and the pores in their vicinity, as motivated by empirical observations from the in-vitro experiments. In the biased-random movement, we compute the probability of movement in each direction (direction-p) depending on the number of neighboring cells at that direction of a cell within its range of attraction, defined as (${L}_{c}$). Also, the probability in each direction can be increased depending on the Euclidean distance between the individual and pores within a specific attraction range known as (${L}_{p}$). In the random movement case, ${P}_{\mathrm{up}}={P}_{\mathrm{down}}={P}_{\mathrm{left}}={P}_{\mathrm{right}}={P}_{\mathrm{forward}}={P}_{\mathrm{backward}}=1/6$, where 6 is the number of directions, and ${P}_{\mathrm{up}}$, ${P}_{\mathrm{down}}$, ${P}_{\mathrm{left}}$ ${P}_{\mathrm{right}}$ , ${P}_{forward}$ , and ${P}_{\mathrm{backward}}$ are the probability of cell movement in the up, down, left and right direction, respectively. In the random-biased case, ${P}_{\mathrm{up}}={P}_{1}$, ${P}_{\mathrm{down}}={P}_{2}$, ${P}_{\mathrm{right}}={P}_{3}$, ${P}_{\mathrm{left}}={P}_{4}$, ${P}_{forward}={P}_{5}$ , and ${P}_{backward}={P}_{6}$, with ${\sum }_{i=1}^{6}{P}_{i}=1$. The probabilities ${P}_{i}$ are computed by scanning and summing the neighboring cells and pore lattice points. More details are described in the Supplementary Material. Finally, each cell moves with more tendency in the direction where higher probability is computed.

### Death

In the simulations, we assumed that the porous structure of the 3D bioprinted scaffold allowed all cells to access nutrients and oxygen, which prevented death due to the lack of such vital materials. However, cells lose their viability if they remained in the stationary phase for more than a specific time defined as ${C}_{d}$, calibrated within a range of values, with the probability of ${P}_{d}$. Table S1 contains the values for all in-silico parameters.

### Statistical analysis

Image J software was utilized to analyze the images. All error bars in the figures and reported data indicated ± SD from at least three repeats (n ≥ 3). The results were reported in the format of mean ± SD. The reported statistics from confocal microscopy images were obtained by averaging the number of cells in at least five distinct points of each structure.

## Supplementary Information

Supplementary Information 1.

Supplementary Information 2.

## Data Availability

The data that support the findings of this study are available from the corresponding author, Dorsa Mohammadrezaei, upon reasonable request.
