# Supplementary material for: Predicting and elucidating the post-printing behavior of 3D printed cancer cells in hydrogel structures by integrating in-vitro and in-silico experiments
Source: Sci Rep. 2023 Jan 21;13:1211. doi: 10.1038/s41598-023-28286-9 (PMC9867702; doi:10.1038/s41598-023-28286-9)
Supplement: Supplementary file 2 — Supplementary Information 2. [file 41598_2023_28286_MOESM2_ESM.docx]

**Predicting and Elucidating the Post-printing Behavior of 3D Printed Cancer Cells in Hydrogel Structures by Integrating In-vitro and In-silico Experiments**

Dorsa Mohammadrezaei^1,*^, Nafiseh Moghimi^1^, Shadi Vandvajdi^1,^ , Gibin Powathil^2^, Sara Hamis^3^, Mohammad Kohandel^1^

^1^ Department of Applied Mathematics, University of Waterloo, Waterloo, Ontario, Canada

^2^ Department of Mathematics, Faculty of Science and Engineering, Swansea University, United Kingdom

^3^ School of Mathematics and Statistics, University of St Andrews, United Kingdom

**Supplementary Material**

**In-vitro studies**


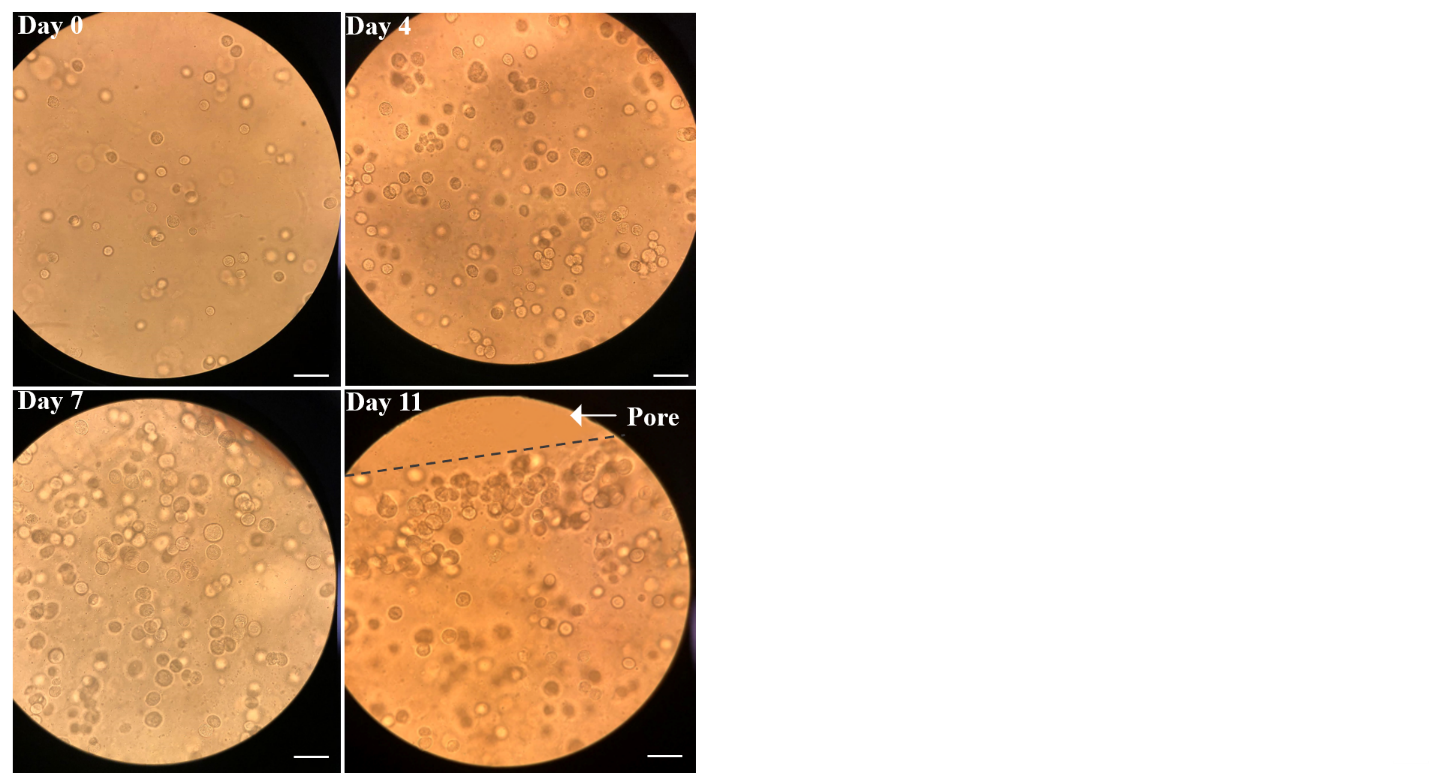
Cells encapsulated in 3D bioprinted structures were monitored by a phase-contrast microscope on days 0, 4, 7 and 11 (Figure S1). Cells show a trend of crawling toward scaffold pores over time, followed by forming clusters.

Figure S1. MDA-MB-231 cells encapsulated in 3D bioprinted structures observed by a phase-contrast microscope on days 0, 4, 7 and 11. Scale bar, 50 μm.


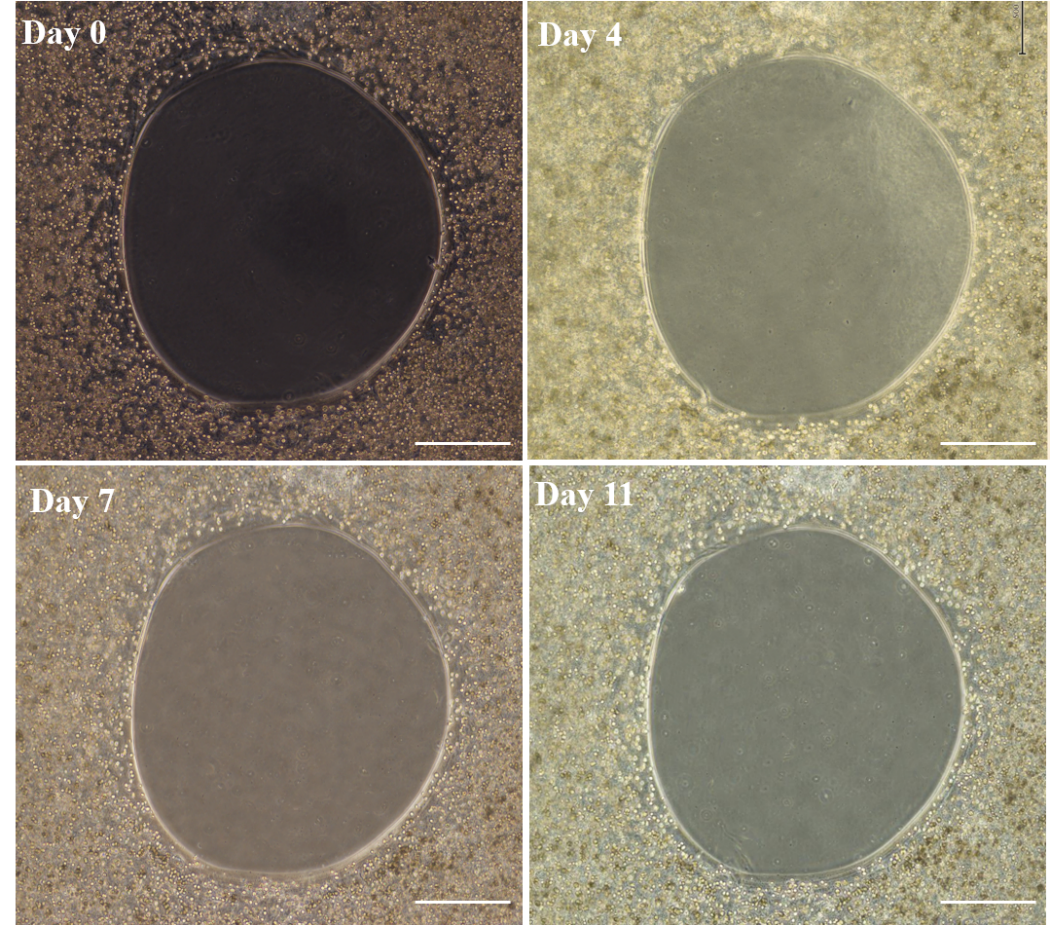


Figure S2. 3D bioprinted constructs observed by a phase-contrast microscope on days 0, 4, 7 and 11. Scale bar, 500 μm.

In addition, as shown in Figure S2, the 3D printed construct could hold its structure and maintain a good form within 11 days. During this period, the pore size remained unchanged, and cells were unable to exit the hydrogel network.

**In-silico studies**

To improve our model description with the purpose of ensuring that enough information is communicated to help model understanding and replication, we used the ODD (Overview, Design concepts and Details) protocol proposed by Grimm et al.^1^

**Model description**

***Overview***

**Purpose**

The purpose of this model is to study the post-printing cellular behaviour of MD-MB-231 cells encapsulated and grown in a 3D hydrogel-based structure fabricated using bioprinting, and investigate the impact of this culture method on cellular proliferation as well as cluster formation over time.

**State Variables and scales**

This model simulates time as discrete, uniform time steps; each time step is 1 hour, and each simulation lasts for 11 days (264 time steps). This model applies a two-dimensional lattice for the purpose of developing a better simulation of the obtained 2D in-vitro data. A subdomain of the porous cell-laden scaffold fabricated using the 3D bioprinting method is simulated in this model, which comprises a square lattice of 190 $\times$ 190$\times$ 30 lattice points, symmetrically consisting of four pores with widths of 50$\times$50$\times$30 lattice points. Each lattice point spans a volume of 1000µ$m^{3}.$Each grid point within the hydrogel can be occupied by a cell or remain vacant, while the grid points in the pores should remain unoccupied. A simulation is initiated by placing a specified initial number of cells (init_c) at random locations on the lattice within the hydrogel. At each time step, cells behave according to a set of stochastic rules that describe cellular processes such as proliferation, movement, and death. Each cell can be characterized by cell-cycle phase and cell-cycle length. Cell-cycle phases include the proliferative phase (G) and non-proliferative or stationary phase (G0), and cell-cycle length is chosen from a normal distribution with a mean of 96 hours and a standard deviation of 6 hours. The mean and standard deviation of this distribution are selected to match the in-vitro results of this study for MDA-MB-231 cells. An overview of state variables, their default values, and experimentally observed data used for their calibration is illustrated in Table 1.

Table s1. Overview of state variables, default values and experimentally observed data used for their calibration.

| **Parameter** | **Value** | **experimentally observed data used for calibration** |
| --- | --- | --- |
| **The total size of the scaffold** | 190$\times190$ $\times$ 30 lattice points | Size of 3D bioprinted scaffold |
| **Pore size** | 50$\times$50$\times$ 30 lattice points | Size of 3D bioprinted scaffold |
| **Time step** | 1 h | - |
| **Initial distribution of cells** | Random | - |
| **Cell phases** | Proliferative phase (G), non-proliferative phase (G0) | Ki-67 test |
| **Cell-cycle length** | Picked from a normal distribution | MTT assay |
| **Mean of cell cycle length distribution (µ)** | 96 h | MTT assay |
| **The standard deviation of cell cycle length distribution (σ)** | 6 h | MTT assay |
| **Probability of random movement** | 0.25 | Microscopic images |
| **Probability of biased-random movement** | 0.75 | Microscopic images |
| $\boldsymbol{m}_{\boldsymbol{C}}$ | 15 h | Microscopic images |
| $\boldsymbol{s}$ | 0 or 1 h | - |
| $\boldsymbol{C}$ | 10,000 cells or lattice points | Proliferation assays |
| $\boldsymbol{L}_{\boldsymbol{C}}$ | 5 lattice points | Microscopic images |
| $\boldsymbol{L}_{\boldsymbol{p}}$ | 10 lattice points | Microscopic images |
| $\boldsymbol{C}_{\mathbf{initial}}$ | 3000 cells | Proliferation assays |
| $\boldsymbol{P}_{\mathbf{0}}$ | 0.5 | Proliferation assays |
| $\boldsymbol{C}_{\boldsymbol{d}}$ | 48 h | Proliferation assays |
| $\boldsymbol{P}_{\boldsymbol{d}}$ | 0.03 | Proliferation assays |

**Process Overview and Scheduling**

Process overview and scheduling are illustrated in Figure 1. At each time step, cells proliferate and divide to create a daughter cell. Also, individual cells move randomly or biasedly and change their positions if there is a free lattice point in their neighbourhood. In our study, cell death occurs under specific conditions.

***Design Concepts***

**Emergence**

The emergent behaviour in this model exists in the cellular proliferation of cancer cells. Indeed, cancer cells encapsulated in the hydrogel network can show different proliferation rates post-bioprinting depending on the initial number of cultured cells; cell movement (biased-random or random) and the direction they move; the position they plant their daughter cells in their neighbourhood; carrying capacity of the scaffold; and accumulation of vital materials.

**Sensing**

In the proliferation process, the parent cells sense the presence of other cells in their neighbourhood, and daughter cells can only be placed in an unoccupied lattice point in the neighbourhood of the parental cell. If all lattice points within a third-order Moore neighbourhood of the parental cell is occupied, no daughter cell will be placed, and the parental cell will enter the G0 phase. Additionally, in the biased-random movement toward neighbouring cells, each cell can access the number of cells within its range of attraction and attempts to move toward the direction in which there are more cells.

**Interaction**

Cells in this model have indirect interactions as the proliferation is assumed to be aborted when the total number of cells reaches the maximum capacity of the scaffold or all neighbouring positions are occupied by other cells. Cells also move towards each other in biased-random movement, and daughter cells are placed in the neighbourhood of parent cells.

**Stochasticity**

Behavioural stochasticity is represented in this simulation. In the initialization step of the simulation, cells in the active phase (G1) are seeded at random positions. Additionally, for proliferation and movement and death processes, there are associated probabilities that cells make certain decisions. For example, in cell movement, cells do not always move randomly and sometimes move in a biased-random manner instead. Whether a cell decides to do a biased-random movement or random movement at any time step is simulated stochastically (using parameters called biased probability and random probability). Cells move every $m_{C}+s$ hours, where $s$ is a random number (0 or 1) in order to avoid the synchronized movement of all cells. The direction of movement is finally selected stochastically depending on the probability computed for each direction. Also, in cell proliferation, each cell is given an individual stochastic doubling-time that is derived from a normal distribution with a determined mean and standard deviation.

***Details***

**Initialization**

The values of parameters utilized in initialization are based on the carried out experiments. Random lattice points are initially occupied by the specific initial density of cells in a two-dimensional lattice. For initial values of parameters, look at table 1. Initialization consists of two steps:

**Setup scaffold**

A scaffold is set in lattice points, such that scaffold[z][n]=1, where there are printed layers of hydrogel, and scaffold[z][n]=0 where there are pores (z and n are representatives of the lattice point coordinates).

**Place Cells in Random Locations**

- Each initial cell is planted randomly in the position (z,n), if and only if scaffold[z][n]=1.
- Check if the new location is free or occupied by a cell. If it is free, a cell is planted in a new random location, and the counter of cell density "no_planted_cells" is incremented by one; otherwise, a different location is tried by redoing the while loop without incrementing the number of planted cells.
- After planting each cell at any position, the status of positions is updated by getting changed from 0 to 1.
- On day 0, the in-vitro results showed that 24±2% of the cells in bioink were damaged and dead due to being exposed to the high pressure during the bioprinting process. However, we did not consider the initial dead cells in the model, and cultured only viable cells on day 0.

**Input**

A subdomain of the porous cell-laden scaffold fabricated using 3D bioprinting method is simulated in this model, which comprises a square lattice of 190 $\times$ 190 $\times$ 30 lattice points, symmetrically consisting of four pores with 50$\times$50 $\times$ 30 lattice points.

**Submodels**

Within each time step, the following processes are defined in this model.

**Cell movement**

In this process, each cell can move in a random way or biased-random manner and change its positions if there is a free lattice point in its neighbourhood. The probability of biased-random movement is considered to be 0.75, and the probability of random movement is equal to 0.25. Cells move every 15 hours known as $m_{c}$, however as all the cells might not be synchronized in their movement, a random number (0 or 1) was introduced to be added to $m_{c}$. The value of the $m_{c}$ parameter was also calibrated using in-vitro data. In the biased movement, the probability in each direction is computed using the number of neighbouring cells at that direction of a cell within $L_{c}=5$ (lattice points) and the Euclidean distance between the individual and pores within $L_{p}=10$.

For example, the probability of movement in up direction is computed similar to the following:

For the range of $L_{p}$ lattice points around each focal cell:

$X_{up}=\sum_{i} \frac{h_{i}}{l_{i}}$ W

For the range of $L_{c}$ lattice points around each focal cell:

$p_{\mathrm{up}}=Y$

Here W is a constant weight, $i$is sum over $L_{p}$lattice points that are above the focal cell, $h_{i}=\left\{ \begin{aligned} 1, & if lattice point is a pore \\ 0, & otherwise \end{aligned} \right.,L_{i}$is the Euclidean distance between the focal cell and lattice point i. $Y$ is the number of neighboring cells in $L_{c}$ lattice The value $W=100$, $L_{c}=5$ (lattice points) and $L_{p}=10$ (lattice points) are calibrated to consider the empirical observation that cells were more desired to move toward pores where more vital materials were found, in comparison to the movement toward other cells. Finally,

$$P_{\mathrm{up}}={{(X}_{up} +p}_{\mathrm{up}}) N$$

Indeed, $X_{up}$ and $p_{\mathrm{up}}$ are the contribution from pores and cells, respectively, and N is normalization constant. Therefore, we end with $P_{\mathrm{up}}$+$P_{\mathrm{down}}$+$P_{\mathrm{right}}+P_{\mathrm{left}}+P_{\mathrm{forward}}+P_{\mathrm{backward}}$=1, and each focal cell is more likely to move in the direction where a higher directional probability is computed.

Other submodels (Cell Proliferation and Cell Death) are described in the Method section. All the parameters in these submodels are calibrated to best fit the experimental data. The animation of cell growth within the hydrogel scaffold in 11 days is illustrated in figure S3.


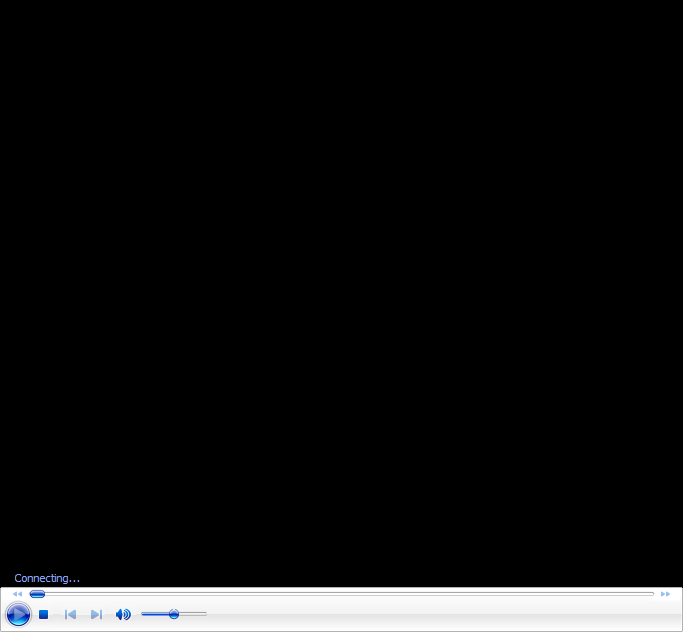


Figure S3. Animation of MDA-MB-231 growth within 3D hydrogel network in 11 days. yellow represents proliferating cells; red represents non-proliferating cells; black represents dead cells.

**Consistency analysis**

Consistency analysis is a technique that is applied to find how many times the simulation should be run before describing our results in order to minimize the uncertainty originating from inherent model stochasticity. This simulation experiment was conducted based on the study performed by Hamis s et al.^2^ To do this, we produced five different groups that each contains $k=20$ distributions of size $n=\{1, 5, 50, 100, 200\}$. We also specified two outputs of interest: X1: the number of living cells on day 5, and X2: the number of proliferating cells on day 5. The purpose of consistency analysis is to determine a distribution size that leads to small statistical significance for these results. In each group, we computed and plotted the maximal Â-measure^3^ for $k=1,2,\ldots, 20$ distributions and for both X1 and X2 separately, as is shown in Figures S4, S5, S6, and S7.


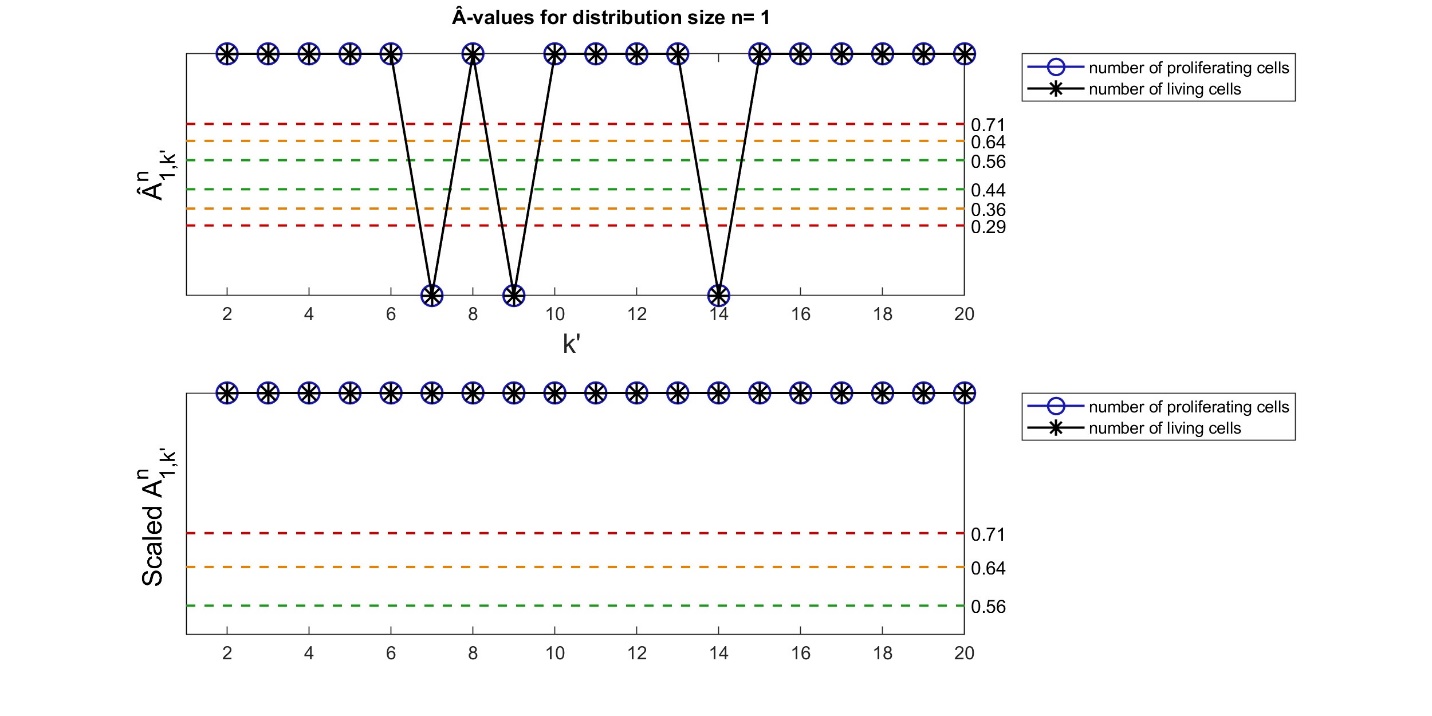


Figure S4. Consistency Analysis, Â-values and scaled Â-values for $n=1$.


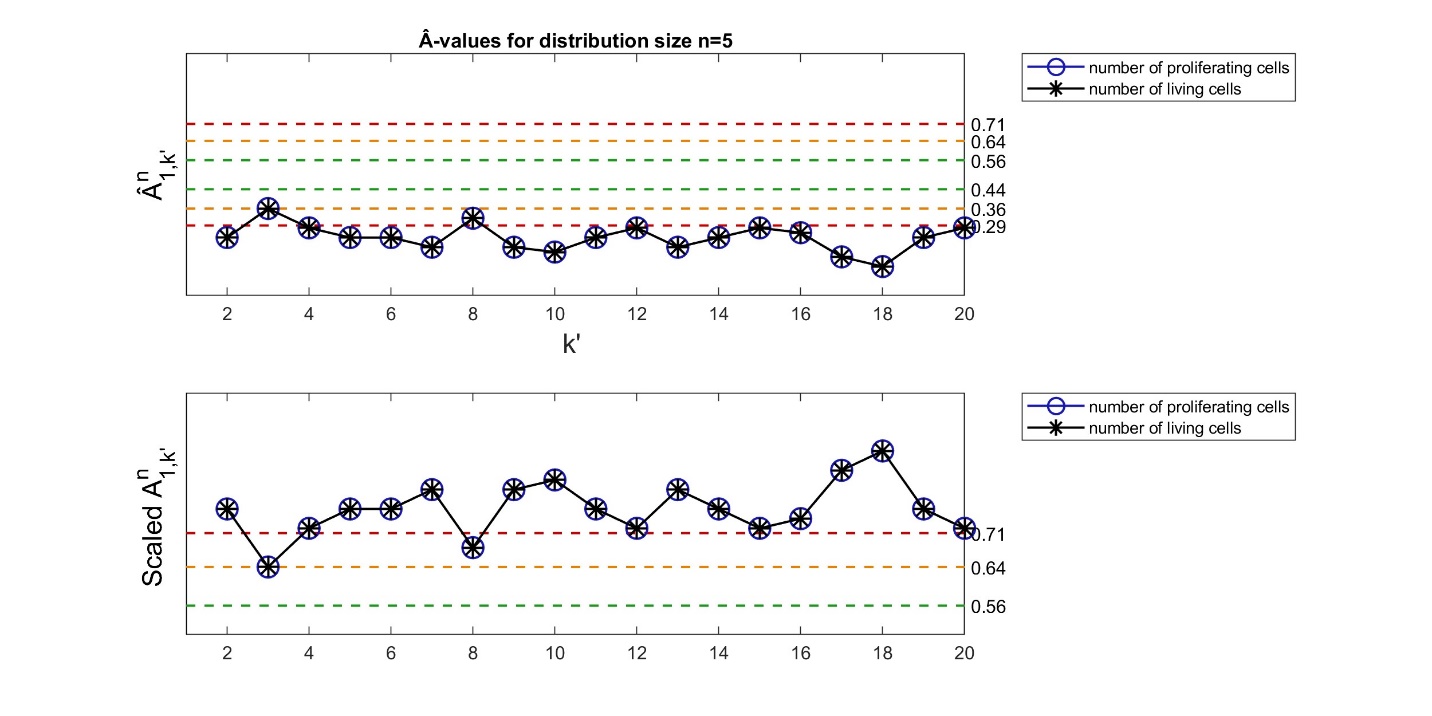


Figure S5. Consistency Analysis, Â-values and scaled Â-values for $n=5$.


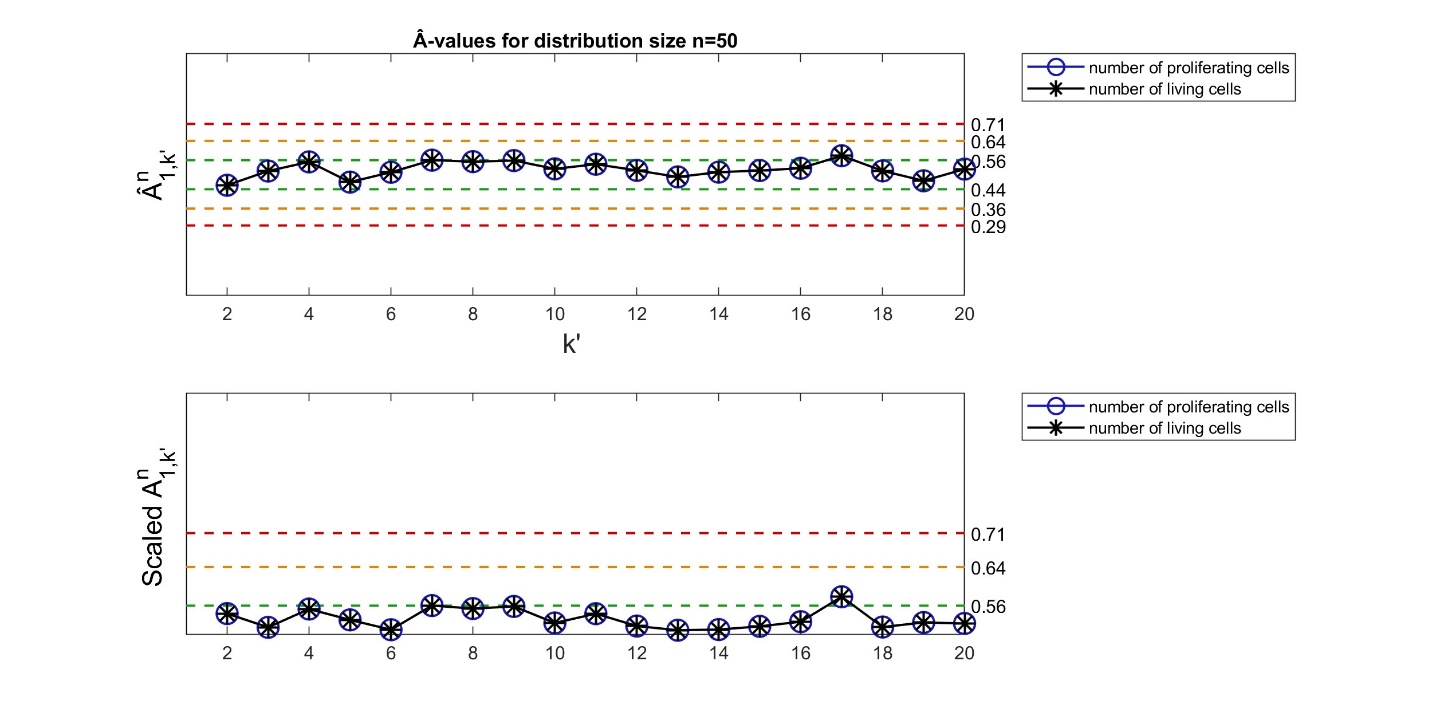


Figure S6. Consistency Analysis, Â-values and scaled Â-values for $n=50$.


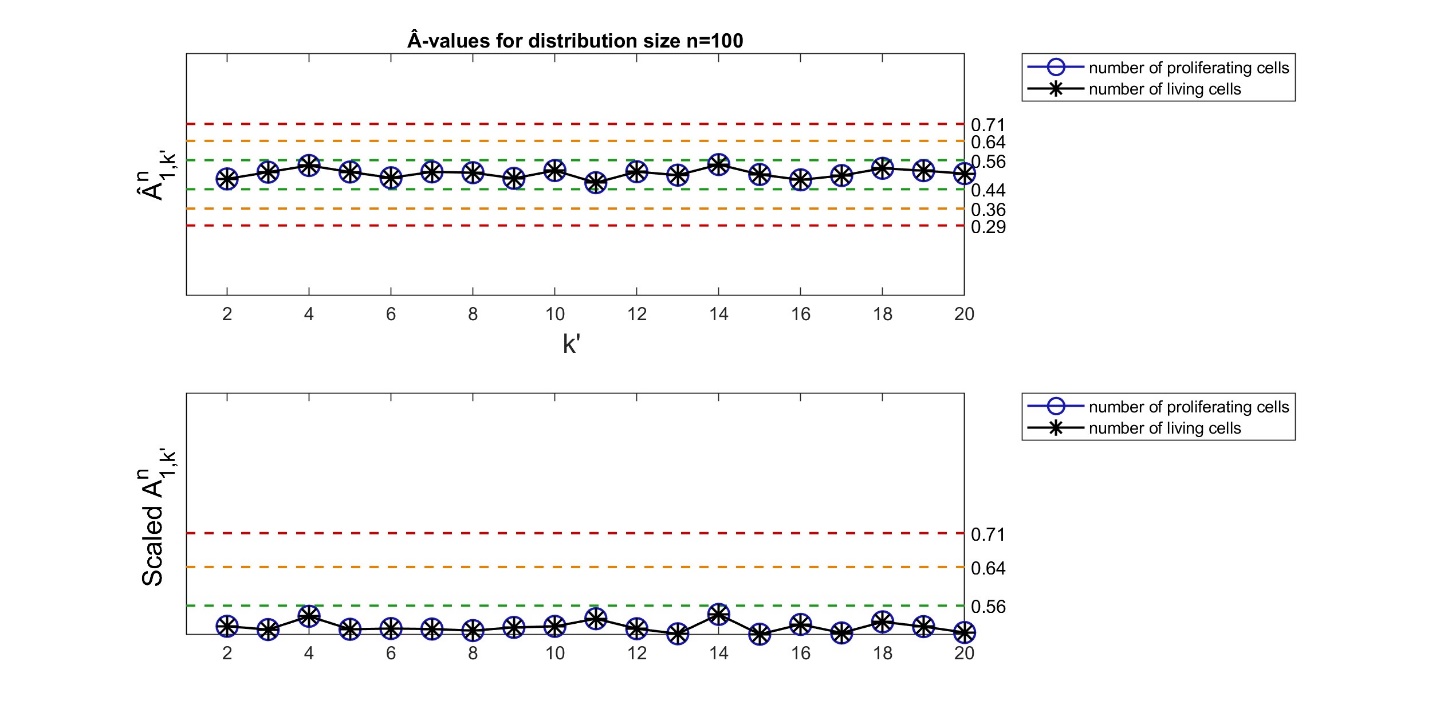


Figure S7. Consistency Analysis, Â-values and scaled Â-values for $n=100$.

The figures above demonstrated that the minimum distribution size for which the statistical significance is considered small (< 0.56) for both outputs of interest is $n=100$. Therefore, all in-silico results are based on an average obtained from 100 simulation runs in this study.

**References**

1. Grimm, V. *et al.* A standard protocol for describing individual-based and agent-based models. *Ecol. Modell.* **198**, 115–126 (2006).

2. Hamis, S., Yates, J., Chaplain, M. A. J. & Powathil, G. G. Targeting Cellular DNA Damage Responses in Cancer: An In Vitro-Calibrated Agent-Based Model Simulating Monolayer and Spheroid Treatment Responses to ATR-Inhibiting Drugs. *Bull. Math. Biol.* **83**, 1–21 (2021).

3. Hamis, S., Stratiev, S. & Powathil, G. G. Uncertainty and sensitivity analyses methods for agent-based mathematical models: An introductory review. *Phys. Cancer, Res. Adv.* 1–37 (2020) doi:10.1142/9789811223495_0001.
